# Prognosis of patients with heart failure and reduced ejection fraction in China

**DOI:** 10.3892/etm.2013.1341

**Published:** 2013-10-11

**Authors:** YU XU, YANAN SHI, ZHONGYU ZHU, CHANGHE CUI, BEI LI, FANG CHEN, DAN LI, SONGHU CHEN, YANG GUO

**Affiliations:** Department of Cardiology, Henan Province People’s Hospital, The People’s Hospital of Zhengzhou University, Zhengzhou, Henan 450003, P.R. China

**Keywords:** heart failure, prognosis, left ventricular ejection fraction, medication status quo

## Abstract

The present study aimed to investigate the 5-year survival and medication status of patients with chronic heart failure (HF) and reduced ejection fraction (HFrEF) in China. This study is a single-center, retrospective study and patients with HF and a left ventricular ejection fraction (LVEF) of ≤45%, were consecutively enrolled. The study population of 685 patients was divided into two groups, namely, LVEF ≤35 (n=371) and LVEF 36–45% (n=314). The patients were followed up for a median of 31 months (range, 8–61 months) and during this period, 24% of patients receiving angiotension-converting enzyme inhibitor/angiotensin receptor blocker (ACEI/ARB) treatment and 23% of those receiving β-blockers reached the maximum tolerated dose. Of the 191 total mortalities (28%), 127 were due to pump failure (19%) and 42 (6%) were sudden deaths. A Cox proportional hazards regression model was used to identify the variables associated with the risk of mortality. Kaplan-Meier curves and log-rank tests were used to compare the survival times of patients in the LVEF ≤35% and LVEF of 36–45% groups. The predictors of all-cause mortality were advanced age, body mass index, New York Heart Association functional class and lack of oral β-blockers at discharge. Patients with HFrEF have poor prognoses in China, particularly those patients with an LVEF of ≤35%. Therefore, cardiologists should strive to improve the prognosis of HF among Chinese patients and focus on the importance of the practical application of HF diagnosis and treatment guidelines.

## Introduction

Heart failure (HF) is a modern epidemic and a significant public health problem. Patients with HF are frequently hospitalized and have a high mortality rate. Regardless of the remarkable advances in diagnosis and therapy over the past decade, the prognosis of patients with HF remains poor, with mortality rates approaching 20% per year (1). With changing epidemiological and socioeconomical developments, the epidemiology characteristics of HF in developing and developed countries are becoming increasingly similar, such that coronary heart disease as HF etiology is increasingly prominent in China (2,3). However, the overall profile and prognosis of patients with HF and reduced ejection fraction (HFrEF) is very limited (4). Most Chinese cardiologists are challenged with the high mortality rate of patients with HFrEF. The present study retrospectively analyzed a cohort of 685 Chinese patients to clarify the overall profile and prognosis of HFrEF. A left ventricular ejection fraction (LVEF) of ≤45% is defined as a significantly reduced LVEF (4).

## Subjects and methods

### Study groups

A total of 748 patients were admitted to the Department of Cardiology, Henan Provincial People’s Hospital (Zhengzhou, China) from June 14, 2007 to January 27, 2012. Patients were diagnosed with HF according to the modified Framingham criteria for HF (5) and an LVEF of ≤45% was determined by echocardiography during hospitalization. Patients were excluded from this study if they had recent acute coronary syndrome, acute viral myocarditis, congenital heart disease or severe heart valve disease. In addition, patients that had other concomitant diseases that are associated with a reduced life expectancy, including malignant tumors, severe hematological system disorders, chronic respiratory failure and end-stage cirrhosis, were excluded. Sixty-three patients (9%) who had incomplete clinical data or were lost during the follow-up period, were also excluded from this study. The study population consisted of 685 patients with HF, divided into two groups: patients with an LVEF of ≤35% (n=371) or an LVEF of 36–45% (n=314). Moreover, if the patient was hospitalized more than once due to HF, only the data from the first hospitalization was analyzed. This study was conducted in accordance with the Declaration of Helsinki and with approval from the ethics committee and the Institutional Review Board of Henan Provincial People’s Hospital (Zhengzhou, China). Written informed consent was obtained from all participants.

### Data extraction

The data relating to the demographic status of patients, including age, gender, body weight, height, place of residence, admission date, cause of admission, background (concurrent) diseases, drug use during hospitalization and drug prescription on discharge, were recorded systematically from the medical records during hospitalization. Body mass index (BMI) was calculated using the following equation: BMI = body weight (kg)/[height (m)]^2^. Moreover, the estimated glomerular filtration rate (eGFR) was calculated as described in a previous study (6) using the following equation: eGFR = 186 × SCr−1.154 × age−0.203 (x 0.742 if female) ml/min/1.73 m^2^.

### Study endpoints

The study endpoints included registration for total mortality and sudden or pump failure death. Mortality was defined as sudden if it occurred within 24 h in the absence of pre-existing progressive circulatory failure or other causes of mortality, as well as if a witnessed death occurred within 60 min of the emergence of new symptoms. Pump failure death was defined as those occurring due to refractory progressive end-stage HF.

### Follow-up

Information regarding the clinical outcome was collected from the patients, dependents of patients or referring physician via telephone interviews, letters or clinical visits.

### Statistical analysis

Statistical analyses were performed using SPSS software, version 13.0 (SPSS, Inc., Chicago, IL, USA). Data are presented as the mean ± standard deviation or median for continuous data and the significance was analyzed using a two-sampled t-test. Categorical variables were described in terms of frequencies and percentages and tested using a Chi-square or a Fisher’s exact test when the theoretical frequency was ≥1 or <5. Cox proportional hazards regression analysis for time of death was used to identify the factors associated with the increased risk of mortality. A forward step method was used to define the final model and the independent predictors of mortality. Results are presented as hazard ratios (HR) and 95% confidence intervals (CI) for each covariate in the model. The Kaplan-Meier survival curves were plotted and the groups were compared using the log-rank test. All P-values were calculated from a 2-tailed test and P<0.05 was considered to indicate a statistically significant difference.

## Results

### Clinical parameters

The baseline characteristics of the 685 patients in this study are shown in Table I. Patients with an LVEF of ≤35% had a significantly higher heart rate (87±19 versus 80±15 bpm, P=0.000), increased incidence of ventricular tachycardia (18 versus 12%, P=0.021), New York Heart Association (NYHA) classes III (41 versus 32%, P=0.017) and IV (30 versus 17%, P=0.000) upon admission, than the patients with LVEFs of 36–45%. Compared to ejection fraction of 36% −45% of the patients, the ejection fraction ≤35% of patients with low serum sodium (140±4 versus 141±5 mmol/l, P=0.049). Furthermore, patients with an LVEF of ≤35% were less likely to be receiving aspirin (62 versus 73%, P=0.003), nitrates (45 versus 54%, P=0.018), statins (32 versus 49%, P=0.000) and clopidogrel (10 versus 23%, P=0.000), but more likely to be taking diuretics (93 versus 85%, P=0.001), digoxin (89 versus 70%, P=0.000), spironolactone (86 versus 78%, P=0.009), coenzyme Q10 (33 versus 23%, P=0.004) and stem cells (13 versus 6%, P = 0.004). In addition, patients with an LVEF of ≤ 35% had a higher incidence rate of left bundle branch block (9% vs. 5%, P=0.041), as well as a larger left ventricular end-diastolic dimension (69±9 versus 64±8 mm, P=0.000).

### Survival analysis

**Figure 1:**
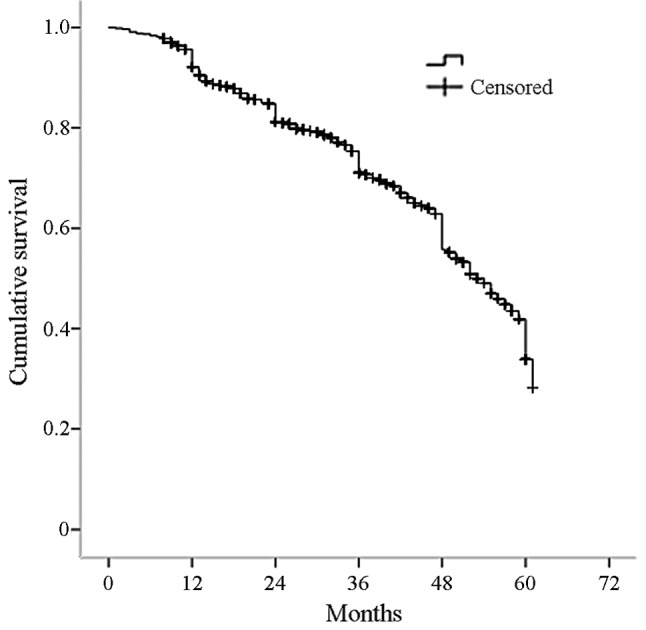
Kaplan-Meier estimated survival curve for the study population.

**Figure 2:**
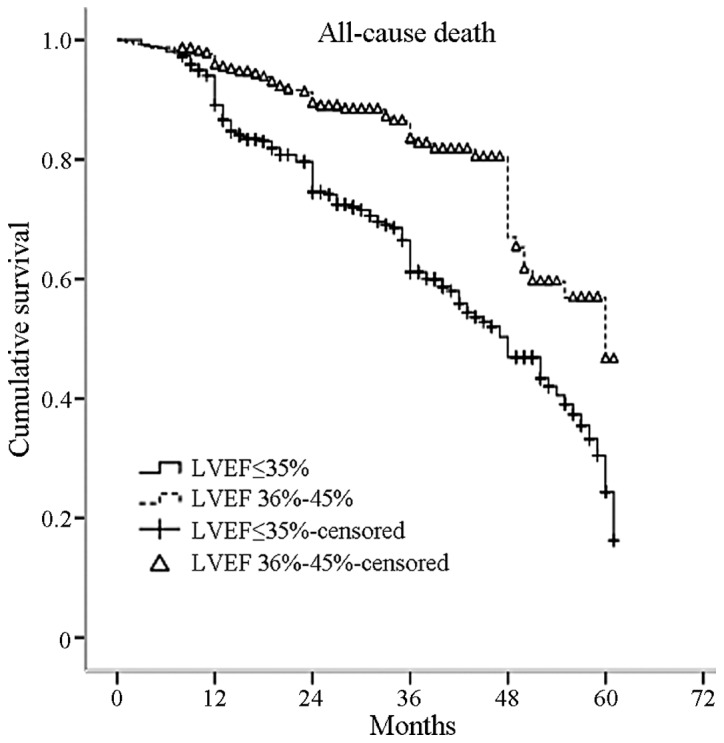
Kaplan-Meier estimated survival curves for the two groups with different left ventricular ejection fraction (LVEF) values (P=0.000 log-rank test).

The patients were followed up for a median of 31 months (range, 8–61 months). A total of 191 mortalities (28%) were recorded, 127 of which were due to pump failure (19%) and 42 were sudden deaths (6%). The all-cause mortality rate was 37% (n=137) among patients with an LVEF of ≤35%, which was significantly higher compared with 17% (n=54) in patients with LVEFs of 36–45% (P=0.000). Pump failure death occurred in 25 and 12% of patients with LVEFs of ≤35 and 36–45%, respectively (P=0.000). Moreover, sudden death occurred in 8 and 4% of patients with LVEFs of ≤35 and 36–45%, respectively (P=0.046). The unadjusted mortality rates from all causes and the Kaplan-Meier estimated survival for the two groups are summarized in Table II. The Kaplan-Meier estimated survival curve for all patients is shown in Fig. 1, with 3, 4 and 5-year survival rates of 71, 56 and 34%, respectively. The Kaplan-Meier estimated survival curves for the two groups are shown in Fig. 2, with 3, 4 and 5-year survival rates of 61, 47 and 25%, respectively, in patients with an LVEF of ≤35% and 83, 66 and 46%, respectively, in patients with an LVEF of 36–45% (P=0.000 log-rank test).

**Figure 3:**
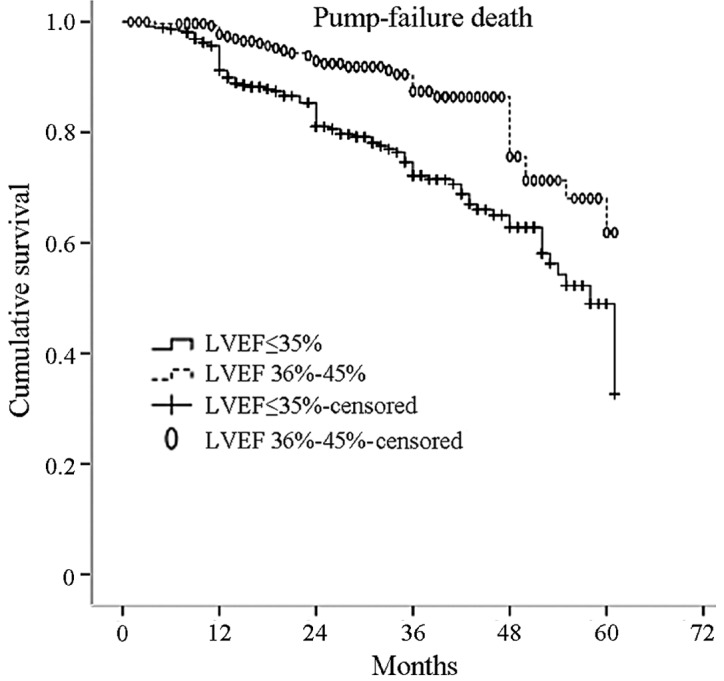
Kaplan-Meier estimated survival curves for the two groups with different left ventricular ejection fraction (LVEF) values (P=0.000 log-rank test).

Kaplan-Meier curves describing the cumulative survival probability of time to occurrence of pump failure death for the two groups, is represented in Fig. 3 (P=0.000 log-rank test).

**Figure 4:**
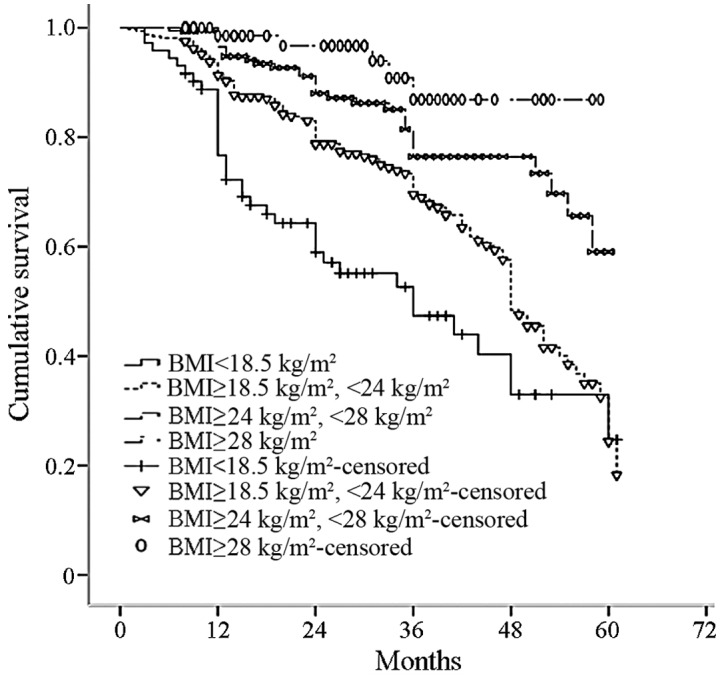
Kaplan-Meier estimated survival curves for four groups with different body mass indices (BMIs) (P=0.000 log-rank test).

Using the recommended BMI classified according to the Working Group of China Obesity (7), the patients were divided into four groups: Low weight (BMI <18.5 kg/m^2^), normal weight (18.5≤BMI<24.0 kg/m^2^), overweight (24.0≤BMI<28.0 kg/m^2^) and obese (BMI ≥28.0 kg/m^2^). The all-cause mortality rate significantly increased with a reduction in BMI (P=0.000; Fig. 4).

**Figure 5:**
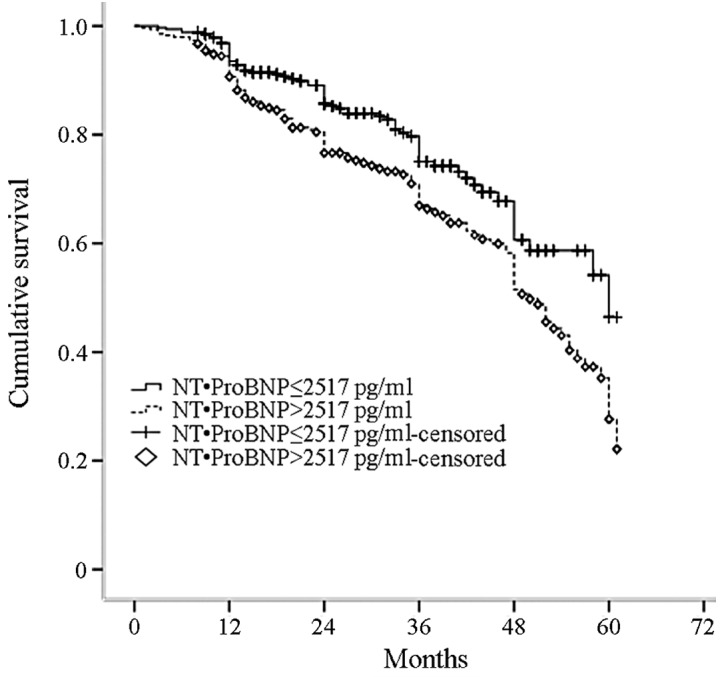
Kaplan-Meier estimated survival curves for two groups with different N-terminal pro-brain natriuretic peptide (NT•proBNP) levels (P=0.003 log-rank test).

The N-terminal pro-brain natriuretic peptide (NT-proBNP) median level was 2,517 pg/ml. The study population was divided into two groups: NT-ProBNP ≤2,517 and >2,517 pg/ml. The all-cause mortality rate significantly increased with an increasing NT-ProBNP median level (P=0.003; Fig. 5).

The predictors of the all-cause mortality rate among the study patients were advanced age and BMI, as well as the lack of oral β-blockers at discharge and NYHA functional class. The results of these multivariate analyses are reported in Table III.

### Medication status quo

During the follow-up period, 24% of the patients who continued to receive angiotension-converting enzyme inhibitor/angiotensin receptor blocker (ACEI/ARB) treatment and 23% of those on β-blockers were taking the maximum tolerated dose. In addition, 17 and 9% were taking the recommended target doses, 47 and 20%, were taking ≥50 to <100% of the target dose, 30 and 36% were receiving ≥25 to <50% of the target dose and 7 and 35% were taking <25% of the target dose.

## Discussion

The present study examined the clinical characteristics and outcomes of Chinese patients with HF and an LVEF of ≤45%. The main findings were as follows: The predictors for all-cause mortality were advanced age, NYHA class, BMI and lack of β-blockers at discharge; the all-cause mortality for the entire study population was 28% during the median follow-up period of 31 months and the 5-year survival rate of patients with HF and LVEFs of ≤35 and 36–45%, were 25 and 46%, respectively; and up to 42% of the patients had ischemic cardiomyopathy and >80% of patients were taking β-blockers, ACEI/ARB, aspirin and diuretics at discharge.

The European Society of Cardiology (ESC) integrated data from 51 countries and demonstrated there are ≥15 million cases of HF among 1 billion patients, as well as a considerable number of patients with asymptomatic heart dysfunction (8). The American Heart Association (AHA) identified that >5 million patients have HF in the United States, a number that continues to increase by 550,000 patients per year^1^. Furthermore, in Japan, the 3-year mortality of patients with HF was reported in 2008 to be 29.2% (9). The situation in Europe is not optimistic, with a 4-year survival rate of only 50% and 40% of the patients admitted to hospitals due to HF are readmitted or die within 1 year of treatment (10,11). In the Framingham Heart study, 75% of men and 62% of women succumbed during the 5-year follow-up time (12). In the current study, the 5-year survival rate of patients was 34%, similar to the findings by Rochester and colleagues in 1991 (13), who noted a 5-year survival rate of 33% among patients with HF during the 10-year follow-up period. The predictors of mortality demonstrated in the current study are consistent with previous reports (9,14). McDonagh *et al*(15) confirmed that, even for asymptomatic patients, the BNP mass concentration was slightly elevated (≥17.9 ng/l), the risk of mortality increased by two-fold (hazard ratio of 2.2) and that LVEF is an important predictor of mortality. The COPERNICUS (16) NT-pro BNP substudy indicated that NT-pro BNP levels above the median (>1,767 pg/ml) on admission were independently associated with an increased risk of all-cause mortality during follow-up. Simultaneously, the meta-analysis conducted by Doust *et al*(17) showed that the BNP mass concentration is closely related to the prognosis of patients with HF. For each additional 100 ng/l increase in BNP mass concentration, a 35% increase in the relative risk of mortality was observed. Additionally, Zamora *et al*(18) assessed the relationship between BMI and survival over a long-term follow-up period of ischemic and non-ischemic HF and concluded that the obesity paradox was only observed in patients with non-ischemic HF. In the MERIT-HF (19) and CIBIS-II (20) trials, β-blockers reduced mortality and sudden cardiac death (SCD), and metoprolol and bisoprolol reduced the all-cause mortality by 34%. A further study (21) demonstrated that carvedilol significantly reduced mortality by 35% (P=0.0014). The results of the three large-scale clinical trials indicate that β-blockers significantly improved the prognosis, showing a significant reduction in total mortality (34–35%) and sudden death (41–45%). It may be suggested that β-blockers are indispensible in the treatment of HF; thus, patients should be prescribed the recommended dose to suppress excessive activation of the sympathetic nervous system, improve cardiac remodeling and reduce sudden death. However, in clinical practice, the application of ACEI/ARB, β-blockers and heart failure treatment guidelines still have a diverse gap; therefore, these therapies should be used widely and consistently. The Chinese Medical Society of Cardiology retrospectively analyzed and compared results of 42 hospitals in 1980, 1990 and 2000 heart failure hospitalization records, and observed that in 2000 the ACEI use ratio was 40.4% in China. The use of β-blockers was <20%, far less than in the United States and Europe in the 1990s (from 60 to 90%). In addition, ARB use was only 4.5% (22). Cao *et al*(23) examined 17 regions in China and demonstrated that 10% of patients received high-dose digoxin (≥0.125 mg/day), 90% of symptomatic patients with chronic HF were prescribed diuretics and 80% of patients received ACEI. The recommended dose application rate was only 2%, the use of β-blockers was 40% and the rate of application of the recommended dose was only 1%. The present study had a small sample size and was a single-center study; therefore, the results may not be applicable to other regions of China.

In China, patients with HFrEF have a poor prognosis, particularly those with an LVEF of ≤35%. Cardiologists should aim to improve the prognosis of HF among Chinese patients and focus on the importance of diagnosis and treatment. Guidelines for the practical application and recommended use of treatment agents should be provided and efforts made to reach the recommended target dose or the maximum tolerated dose in patients.

## Figures and Tables

**Table I:** Clinical characteristics of the study patients.

Parameter	All patients (n=685)	LVEF ≤35% (n=371)	LVEF 36–45% (n=314)	P-value
Male	462 (67)	255 (69)	207 (66)	0.434
Age (year)	57±16	56±16	59±15	0.032
BMI (kg/m^2^)	24±4	23±4	24±4	0.004
SBP (mmHg)	124±21	122±21	128±21	0.000
DBP (mmHg)	80±13	79±14	81±13	0.104
HR (beats per min)	84±18	87±19	80±15	0.000
Hemoglobin (g/l)	128±19	129±19	126±19	0.012
ALT (U/l)	66±272	83±350	46±127	0.066
AST (U/l)	60±379	78±506	38±107	0.136
Uric acid (μmol/l)	416±134	422±137	409±129	0.206
Serum sodium (mmol/l)	140±5	140±4	141±5	0.049
Ischemic cardiomyopathy	287 (42)	123 (33)	164 (52)	0.000
Atrial fibrillation	121 (18)	63 (17)	58 (19)	0.610
Hypertension	223 (33)	95 (26)	128 (41)	0.000
Diabetes mellitus	118 (17)	60 (16)	58 (19)	0.427
Smoking	190 (28)	100 (27)	90 (29)	0.619
Ventricular tachycardia^a^	102 (15)	66 (18)	36 (12)	0.021
ICD implant	15 (2)	11 (3)	4 (1)	0.132
CRT implant	23 (3)	14 (4)	9 (3)	0.511
Stem cell	68 (10)	48 (13)	20 (6)	0.004
KD stage				
1 (≥90)	256 (37)	140 (38)	116 (37)	0.831
2 (60–89)	293 (43)	163 (44)	130 (41)	0.504
3 (30–59)	124 (18)	62 (17)	62 (20)	0.304
4 (15–29)	6 (1)	2 (1)	4 (1)	0.421
5 (<15)	6 (1)	4 (1)	2 (1)	0.693
NYHA class				
II	267 (39)	107 (29)	160 (51)	0.000
III	253 (37)	152 (41)	101 (32)	0.017
IV	165 (24)	112 (30)	53 (17)	0.000
Medications at discharge				
ACE inhibitor/ARB	569 (83)	309 (83)	260 (83)	0.866
β-blockers	555 (81)	302 (81)	253 (81)	0.783
Digoxin	550 (80)	331 (89)	219 (70)	0.000
Diuretics	609 (89)	343 (93)	266 (85)	0.001
Nitrates	333 (49)	165 (45)	168 (54)	0.018
Spirolactone	563 (82)	318 (86)	245 (78)	0.009
Aspirin	458 (67)	230 (62)	228 (73)	0.003
Statins	272 (40)	118 (32)	154 (49)	0.000
Clopidogrel	110 (16)	37 (10)	73 (23)	0.000
Coenzyme Q10	196 (29)	123 (33)	73 (23)	0.004
Sinus rhythm	544 (79)	296 (80)	248 (79)	0.796
Conduction block				
LBBB	50 (7)	34 (9)	16 (5)	0.041
RBBB	31 (5)	16 (4)	15 (5)	0.771
LVEF (%)	34±7	28±6	40±2	0.000
LVEDD (mm)	68±9	69±9	64±8	0.000

Data are presented as mean ± standard deviation or as a frequency and percentage in parentheses. BMI, body mass index; SBP, systolic blood pressure; DBP, diastolic blood pressure; HR, heart rate (evaluated or measured at admission); ALT, alanine aminotransferase; AST, aspartate aminotransferase; ICD, implantable cardioverter-defibrillator; CRT, cardiac resynchronization therapy; eGFR, estimated glomerular filtration rate; CKD, chronic kidney disease; NYHA, New York Heart Association; ACE, angiotension-converting enzyme; ARB, angiotensin receptor blocker; LBBB, left bundle branch block; RBBB, right bundle branch block; LVEF, left ventricular ejection fraction; RBBB, right bundle branch block; LVEF, left ventricular ejection fraction; LVEDD, left ventricular end-diastolic dimension.

a Including sustained and non-sustained ventricular tachychardia.

**Table II:** Unadjusted all-cause mortality and Kaplan-Meier estimated survival rates.

Survival rates (%)	All patients (n=685) (%)	LVEF ≤35% (n=371) (%)	LVEF 36–45% (n=314) (%)	P-value
All-cause mortality	191 (28)	137 (37)	54 (17)	0.000
3-year survival	71	61	83	0.000^a^
4-year survival	56	47	66	-
5-year survival	34	25	46	-

a Log-rank test between the two groups of the Kaplan-Meier estimated survival. LVEF, left ventricular ejection fraction.

**Table III:** Predictors of all-cause mortality after Cox analyses.

Parameter	Hazard Ratio (95% CI)	P-value
Age (per increased 1 year)	1.03 (1.02–1.04)	0.000
NYHA (per increased 1 class)	1.59 (1.32–1.92)	0.000
β-blockers at discharge	0.69 (0.50–0.95)	0.021
BMI	0.58 (0.48–0.72)	0.000
LVEF 36–45%	0.52 (0.38–0.71)	0.000

NYHA, New York Heart Association; BMI, body mass index; LVEF, left ventricular ejection fraction; CI, confidence interval.
